# Supplementary material for: Disentangling degradation pathways of narrow bandgap lead-tin perovskite material and photovoltaic devices
Source: Nat Commun. 2025 Jul 1;16:5450. doi: 10.1038/s41467-025-58489-9 (PMC12219749; doi:10.1038/s41467-025-58489-9)
Supplement: Supplementary file 1 — Supplementary Information [file 41467_2025_58489_MOESM1_ESM.pdf]

Supplementary Information

Disentangling Degradation Pathways of Narrow Bandgap Lead-Tin Perovskite Material and Photovoltaic Devices

*Florine Rombach,[1] Akash Dasgupta,[1] Manuel Kober-Czerny,[1] Heon Jin,[1] James M. Ball,[1] Joel A. Smith,[1] Michael D. Farrar,[1] Henry J. Snaith[1]**

[1]Department of Physics, University of Oxford, Clarendon Laboratory, Parks Road, Oxford, OX1 3PU, UK

Email: henry.snaith@physics.ox.ac.uk

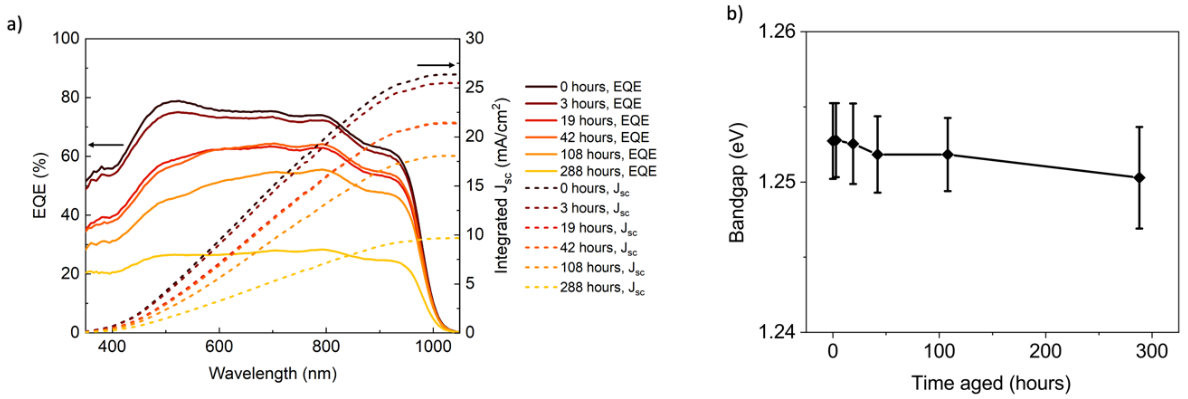

Figure S1. External quantum efficiency (EQE) measurements and extracted bandgap. a) EQE measurements of a typical ITO/PEDOT:PSS/$FA_{0.83}Cs_{0.17}Pb_{0.5}Sn_{0.5}I_3$/PCBM/BCP/Cr/Au device after various periods of encapsulated aging under open circuit, 65 °C, and simulated full spectrum sun light (76 mW cm^{-2}) irradiance conditions. b) Change in the bandgap of devices over time, extracted from measurements in a) by $(E*EQE)^2$ gradient.[1] The mean is plotted with error bars showing the standard deviation (n=4).

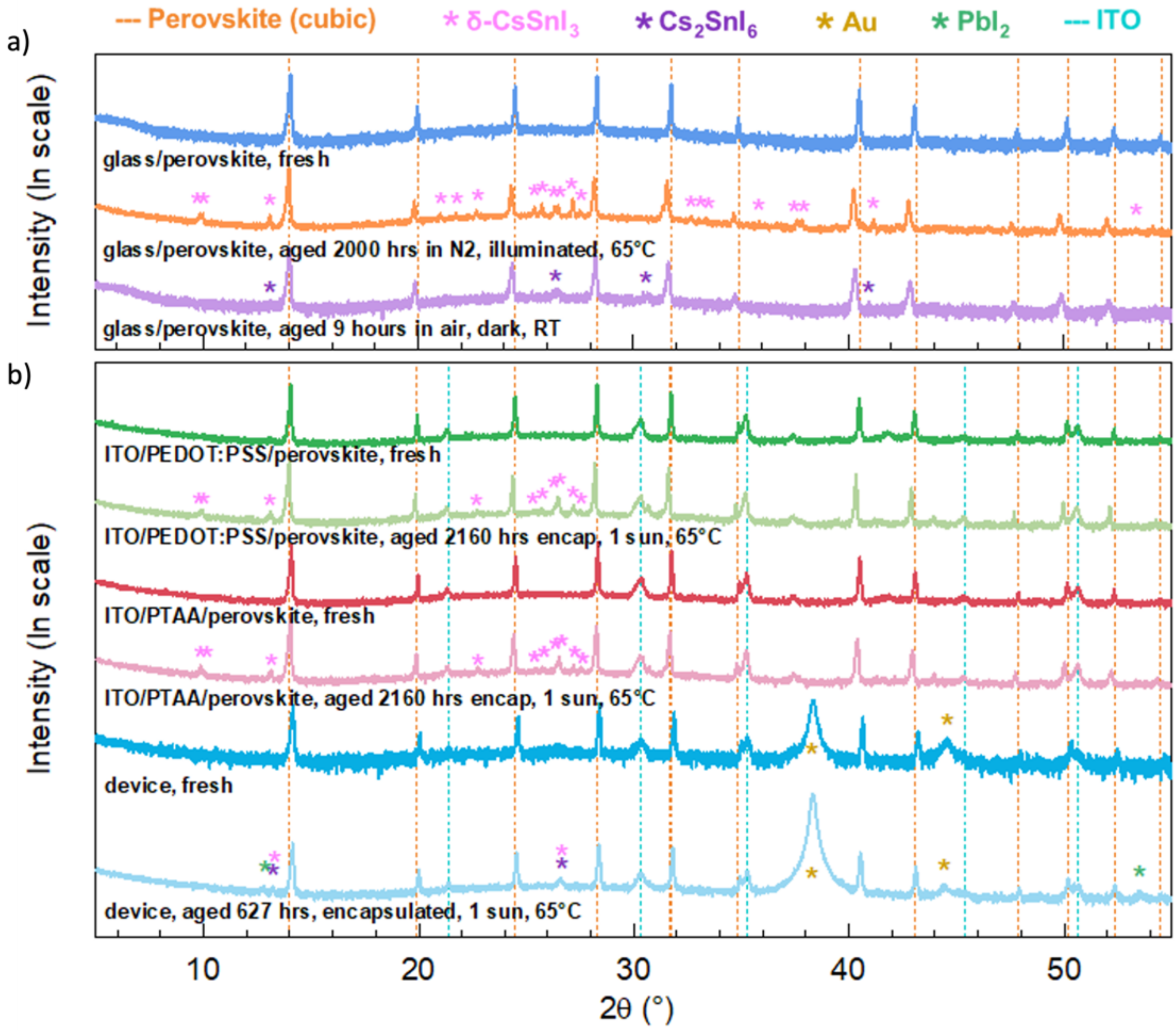

Figure S2. XRD measurements of $FA_{0.83}Cs_{0.17}Pb_{0.5}Sn_{0.5}I_3$ films deposited on a) glass or b) ITO/HTLs, and ITO/PEDOT:PSS/$FA_{0.83}Cs_{0.17}Pb_{0.5}Sn_{0.5}I_3$/PCBM/BCP/Cr/Au devices, aged at 65 °C and simulated full spectrum sun light (76 mW cm^{-2}) irradiance for various periods of time under ambient, ambient encapsulated, or N_2 conditions at open circuit. Films that were encapsulated during aging were measured by cutting the glass edges of the sample to remove the encapsulation glass and epoxy.

Table S1. Unit cell volume of the cubic perovskite cell fitted to PbSn-perovskite films and device before and after aging at 65 °C and simulated full spectrum sun light (76 mW cm^{-2}) irradiance.

**Stack *(aging condition)***	**Unit cell volume before aging [Å^3]**	**Unit cell volume after aging (aging time) [Å^3]**
ITO/PEDOT:PSS/ $FA_{0.83}Cs_{0.17}Pb_{0.5}Sn_{0.5}I_3$/PCBM/BCP/Cr/Au *(encapsulated, 65°C, 0.76 suns)*	**248.76 ± 0.20**	**250.28 ± 0.01** (627 hours)
ITO/PEDOT:PSS/ $FA_{0.83}Cs_{0.17}Pb_{0.5}Sn_{0.5}I_3$ *(encapsulated, 65°C, 0.76 suns)*	**250.18 ± 0.08**	**252.58 ± 0.05** (2160 hours)
ITO/PTAA/Al_2O_3 NPs/ $FA_{0.83}Cs_{0.17}Pb_{0.5}Sn_{0.5}I_3$ *(encapsulated, 65°C, 0.76 suns)*	**250.39 ± 0.06**	**252.23 ± 0.06** (2160 hours)
Glass/Al_2O_3 NPs/ $FA_{0.83}Cs_{0.17}Pb_{0.5}Sn_{0.5}I_3$ *(unencapsulated ambient, room temperature)*	**250.16 ± 0.10**	**253.05 ± 0.02** (9 hours in air)
Glass/Al_2O_3 NPs/ $FA_{0.83}Cs_{0.17}Pb_{0.5}Sn_{0.5}I_3$ *(unencapsulated in N_2, 65°C, 0.76 suns)*	**250.39 ± 0.10**	**254.39 ± 0.07** (2000 hours)

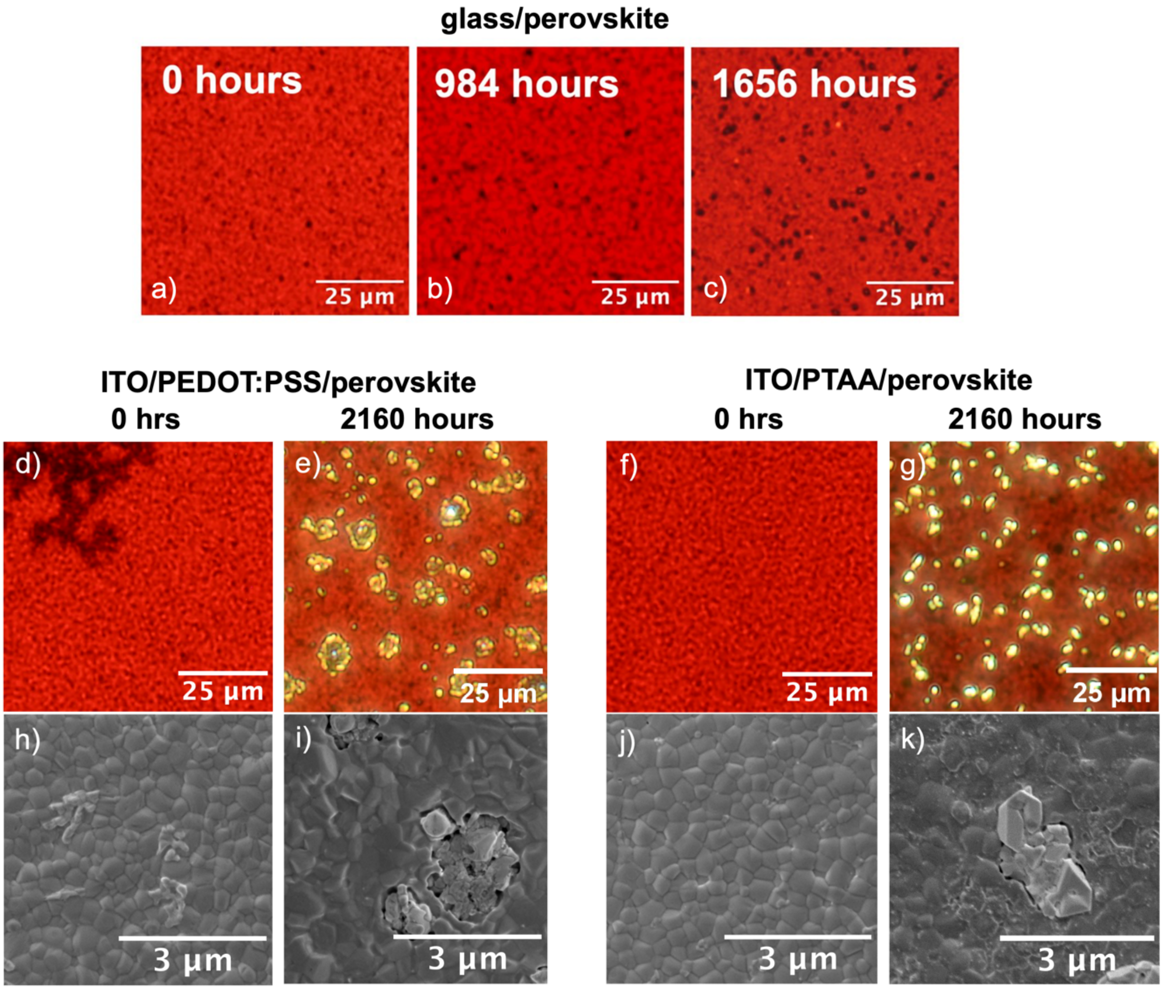

Figure S3. Images of the surface of lead-tin perovskite films after various periods of encapsulated aging under 65 °C and simulated full spectrum sun light (76 mW cm^{-2}) irradiance. Optical microscope (OM) images of the surface of lead-tin perovskite films deposited on glass a) for fresh samples, b) after 984 hours of aging, and c) after 1656 hours of aging, showing the appearance of small yellow regions only beyond 1000 hours of aging. OM images of the surface of lead-tin perovskite films deposited on ITO/PEDOT:PSS d) at 0 hours and e) after 2160 hours of aging, and deposited on PTAA f) at 0 hours and g) after 2160 hours of aging. SEM images of the surface of lead-tin perovskite films deposited on ITO/PEDOT:PSS h) at 0 hours and i) after 2160 hours of aging, and deposited on PTAA j) at 0 hours and k) after 2160 hours of aging.

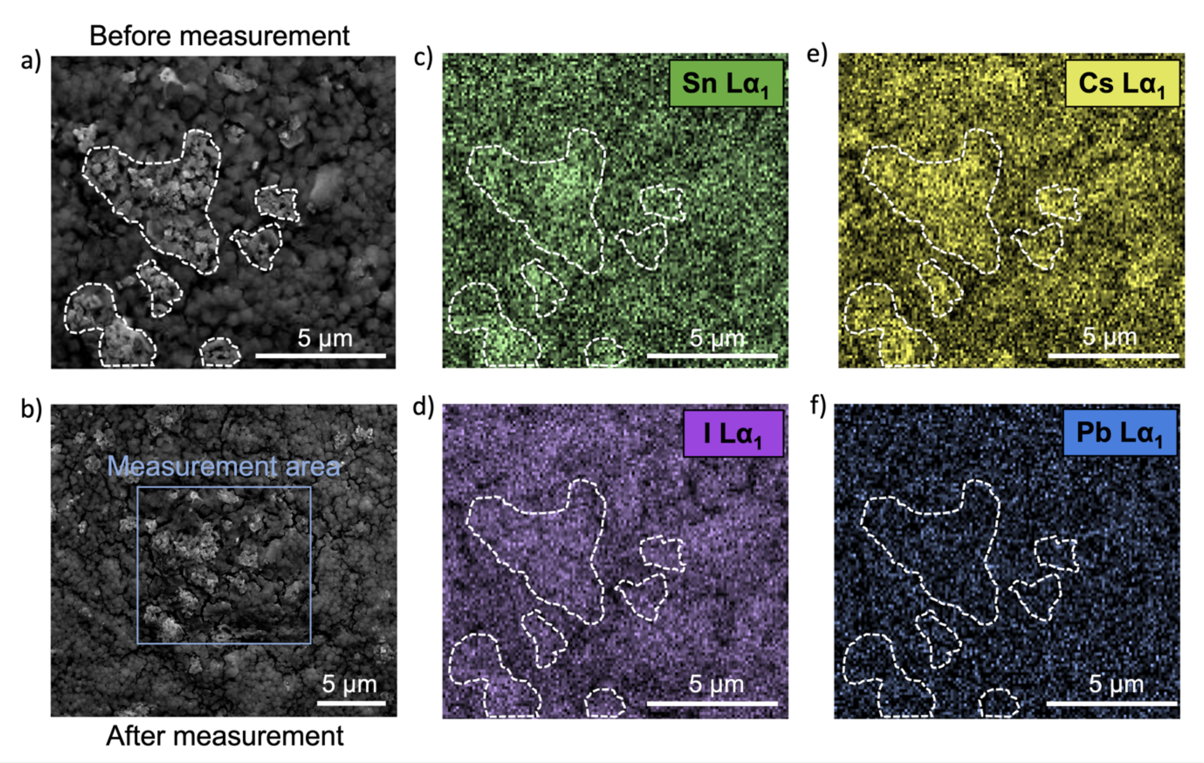

Figure S4. Energy-dispersive X-ray scanning electron microscopy (EDX-SEM) analysis of the surface of lead-tin perovskite films on glass, after 2592 hours of encapsulated aging under 65 °C and simulated full spectrum sun light (76 mW cm^{-2}) irradiance. a) SEM image of the area prior to measurement (new crystallites are marked with white dashed lines) and b) after 7 minutes of measurement, showing some grain boundary decomposition but no heavy degradation. Elemental maps using the Lα_1 spectral line for c) Sn, d) I, e) Cs and f) Pb, with a 5 µm scalebar.

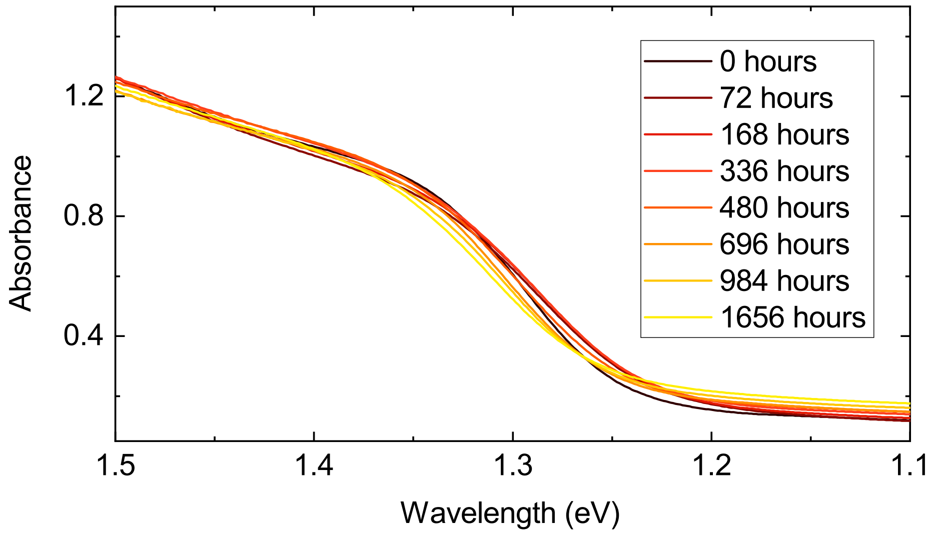

Figure S5. Absorbance of encapsulated lead-tin perovskite films on glass after various periods of encapsulated aging under 65 °C and simulated full spectrum sun light (76 mW cm^{-2}) irradiance.

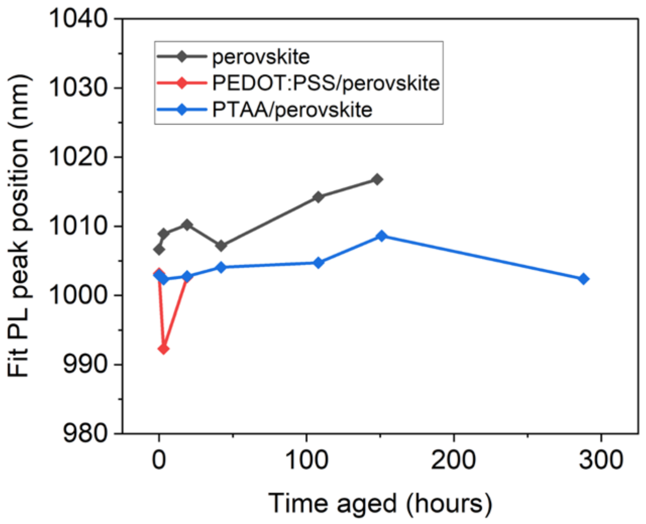

Figure S6. Photoluminescence (PL) peak positions extracted from gaussian fits of the PL signal of perovskite films deposited on glass, glass/PEDOT:PSS or glass/PTAA. PL signal for PEDOT:PSS/perovskite films became too noisy to extract the PL peak position after 19 hours of aging.

Supplementary Note 1: Dependence of photoconductivity of excitation fluence

To calculate the sum of mobilities from the measured TPC decays, we need to estimate the value of the conductivity immediately after the laser pulse. The maximum conductivity measured (which normally occurs 20-40 ns later) is a significant underestimate of this, as during this time free charge carriers have already undergone significant recombination and have also been removed from the perovskite by charging of the electrodes.[2] In this paper, we partially correct this by extrapolating a stretched mono-exponential fit of the photoconductivity back to t_0. However, this only corrects for the charge carriers removed by non-radiative recombination.

To probe the effect of this, we measured TPC decays at 9 different fluences and plotted the extracted sums of mobilities in Figure S7. In all samples we see a decrease in the sum of mobilities with higher excitation fluence, as previously observed by other.[3] This is consistent with a stronger underestimation of the t_0 conductivity at higher excitation fluences, as higher order recombination mechanisms become more dominant. We hence select the maximum sum of mobilities calculated over all excitation fluences as the 'real' mobility.

Interestingly, the dark conductivity also shown significant fluence dependence in some of our samples. Unlike the sum of mobilities extracted from the photoconductivity, dark conductivity increases with fluence. This could be caused either by the delayed release of charge carriers after excitation (for example by de-trapping from long-lived trap states or capacitive effects[4]) and/or by a long-lived increase in mobility as a result of photoexcitation. To minimize the impact of this effect, we use the dark conductivity measured with the lowest excitation fluence to determine p_0.

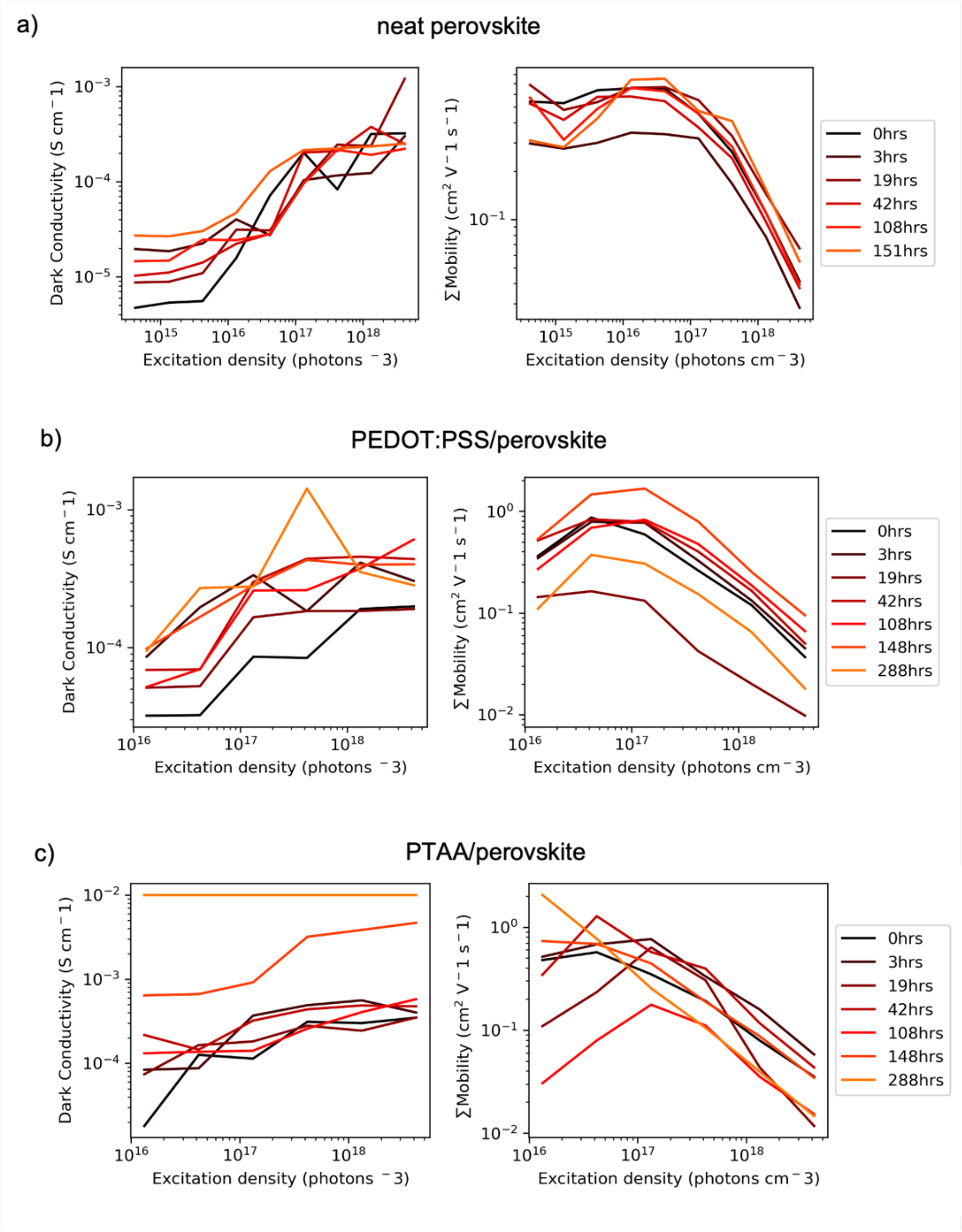

Figure S7. Dark conductivity and sum of mobilities extracted from time-resolved photoconductivity (TPC) traces at a range of excitation densities. Dark conductivity and sum of mobilities of a) a glass/perovskite sample (referred to as 'batch 1' in the main text), b) a glass/PEDOT:PSS/perovskite sample, and c) a glass/PTAA/Al_2O_3 NPs/perovskite sample.

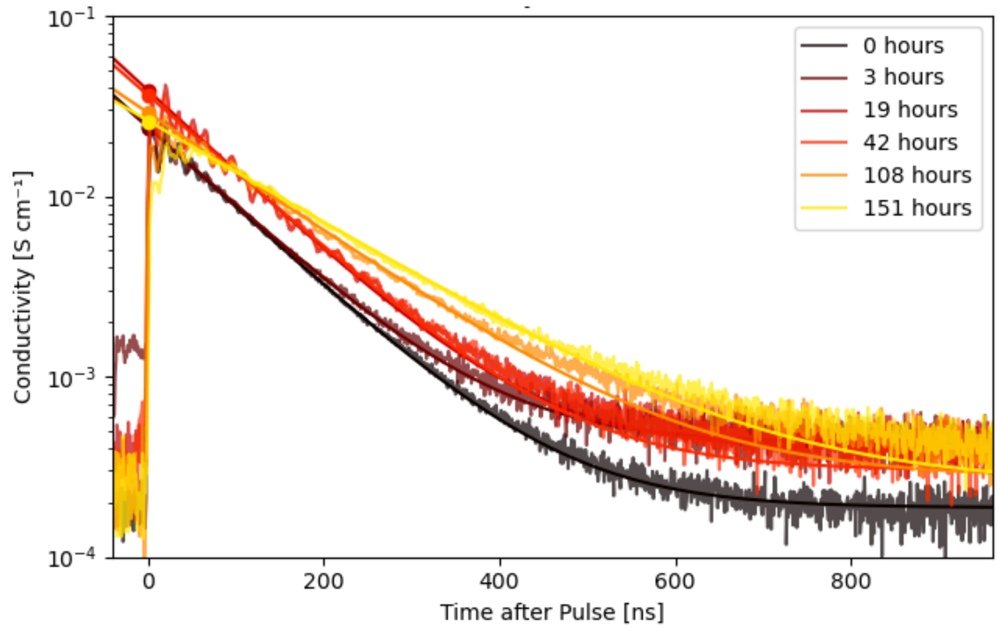

Figure S8. Example TPC transients of lead-tin perovskite films on glass, after pulsed excitation at 10 Hz with 3.74 ns pulses, measured after various periods of aging under 65 °C and simulated full spectrum sun light (76 mW cm^{-2}) irradiance. Various fluences were measured at each aging step, and the fluence resulting in the highest conductivity at t_0 was selected and displayed here (3.0 x 10^4 nJ cm^{-2}). Solid lines represent a fit to a stretched monoexponential decay, which is extrapolated to t = 0 to extract the maximum photoconductivity (solid dot).

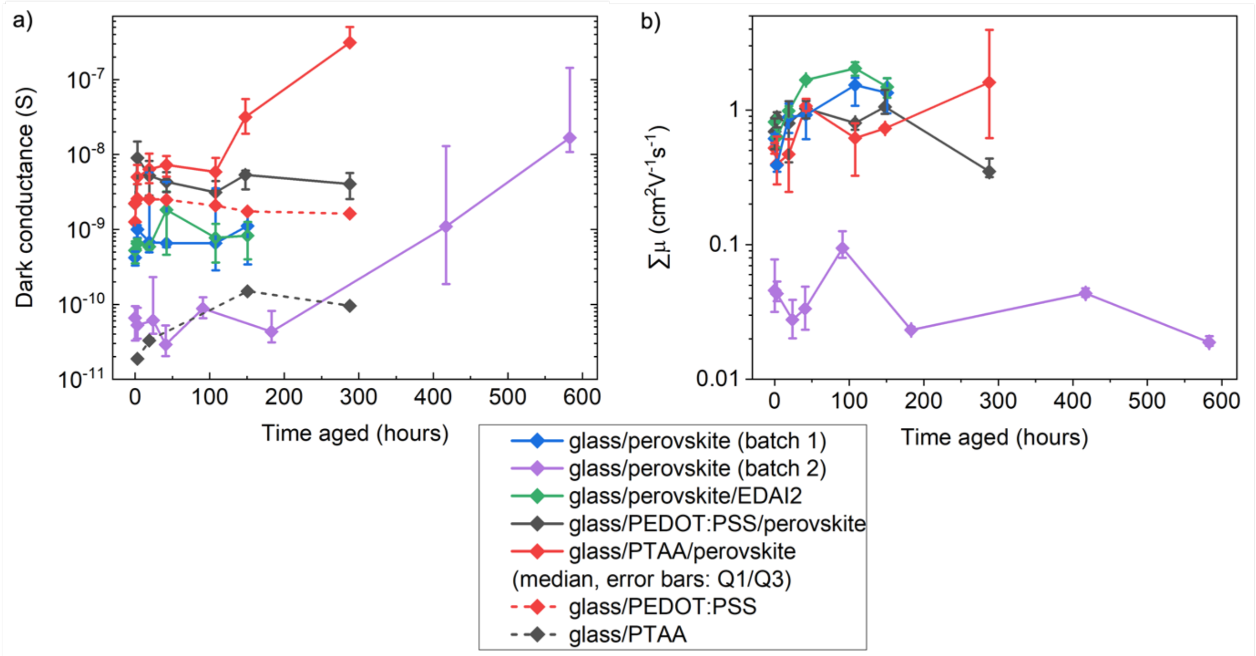

Figure S9. Dark conductance and sum of mobilities of various thin film samples, extracted from TPC traces. a) Dark conductance measured laterally across 300 µm channels on lead-tin perovskite films (660 nm thickness) deposited on glass or thin layers of PEDOT:PSS (diluted 1:6 with IPA) or undoped PTAA (1.5 mg ml^{-1}). Conductance of a film of PEDOT:PSS (diluted 1:6 with IPA) and PTAA (1.5 mg ml^{-1}) deposited on glass measured using the same technique is also shown. b) Sum of mobilities extracted from the conductance shown in a. Measurements were performed after various periods of encapsulated aging under 65 °C and simulated full spectrum sun light (76 mW cm^{-2}) irradiance. Batch 2 of neat perovskite samples shows somewhat worse mobility, likely due to variability in glovebox atmosphere/processing conditions. For all panels, the median is plotted with error bars representing Q1/Q3 (n = 4 for batch 1, n = 2 for $EDAI_2$ passivated, n=3 for batch 2, n=4 for perovskite on PEDOT:PSS or PTAA samples, n=1 for both neat HTL samples).

Supplementary Note 2: Change in PLQE with Fermi level

To confirm whether the trend we observed in the intensity-dependent PLQE of glass/PTAA/perovskite samples can indeed be explained by an increase in background hole density, we performed PLQE simulations. The system modelled is described in the methods. The Fermi level was varied from 0.05-0.5 eV (from the CB), and the single deep electron trap density was varied from 0 for figure S10a to 10^{15} cm^{-3} for figure S10b. All other parameters were kept constant (Table S2).

We find that a downshift in Fermi level along with the presence of non-radiative recombination, as in figure S10b, can indeed explain the trend we observe in the intensity-dependent PLQE measurements of glass/PTAA/perovskite samples (Figure 2c in the main text).

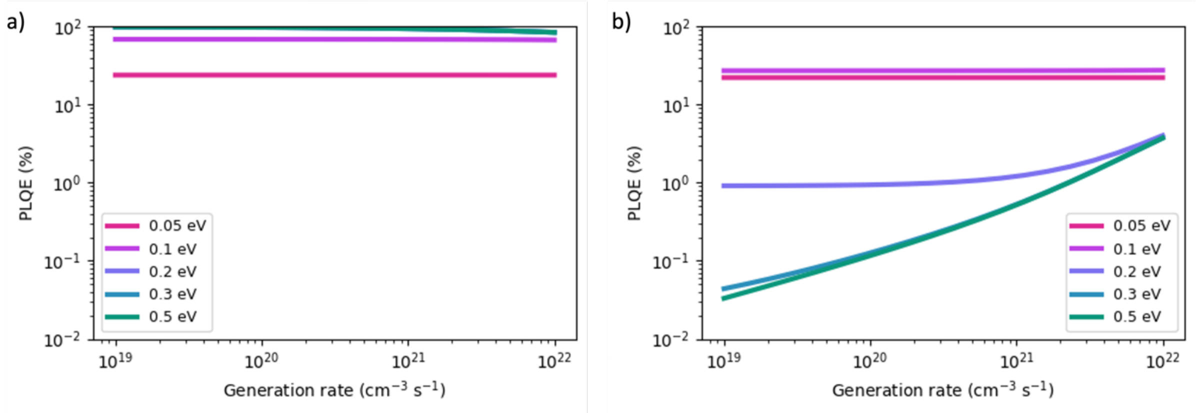

Figure S10. PLQE of perovskite films, simulated as a function of excitation fluence at a range of Fermi level positions a) with no trap-assisted recombination and b) with trap-assisted recombination.

Table S2. Parameters used for PLQE simulations.

**Parameter**	**Symbol**	**Units**	**Value**
Charge carrier density (electron/hole/trap occupancy)	n, p, n_t	cm^{-3}	calc.
Fermi level	E_F	eV	0.3
Excitation fluence	P_{exc}	cm^{-3}	var
Auger recombination coefficient	$k_{aug,n/p}$	cm^6s^{-1}	1×10^{-28}
External radiative band-to-band recombination coefficient	$k_{rad,ext}$	cm^3s^{-1}	5×10^{-12}
Conduction band/valence band energy	E_{CB}, E_{VB}	eV	0, 1.25
Conduction band/valence band density of states	N_C, N_V	cm^{-3}	6.98×10^{18}, 2.49×10^{18}
Effective mass of electron/hole	m_e, m_h	-	0.2, 0.2
Electron/hole capture coefficient	$\beta_{n/p}$	cm^3s^{-1}	10^{-9}, 10^{-9}
Trap energy	E_T	eV	0.3
Trap density	N_t	cm^{-3}	0 or 10^{15}

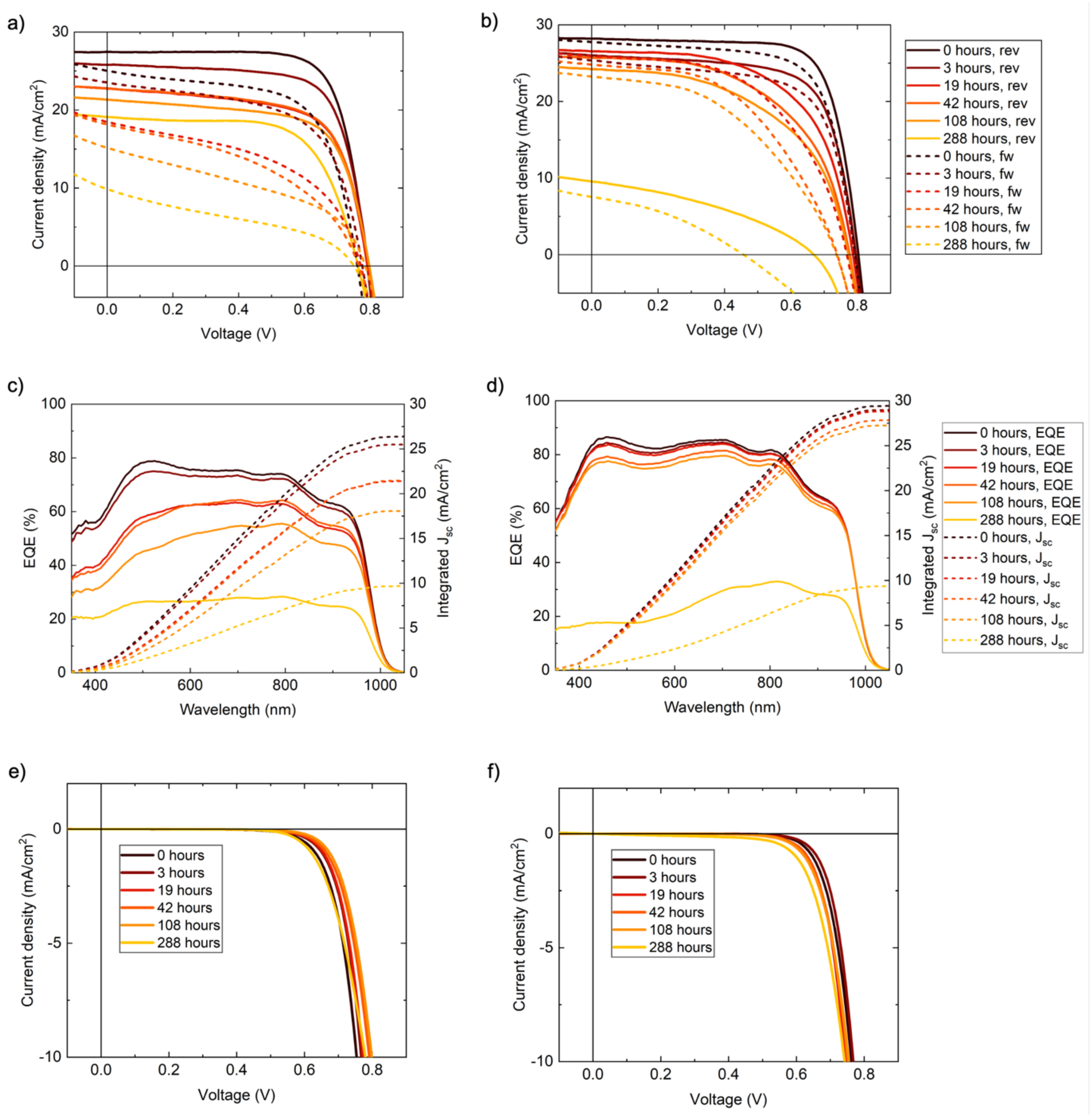

Figure S11. Current density-voltage (J-V) and EQE characteristics of champion lead-tin perovskite ITO/HTL/$FA_{0.83}Cs_{0.17}Pb_{0.5}Sn_{0.5}I_3$/PCBM/BCP/Cr/Au solar cells after various periods of encapsulated aging under 65 °C and simulated full spectrum sun light (76 mW cm^{-2}) irradiance under open circuit conditions. J-V curves measured under AM1.5 100 mW cm^{-2} simulated sun light for devices using a) PEDOT:PSS HTLs, b) PTAA HTLs, EQE curves for devices using c) PEDOT:PSS HTLs, d) PTAA HTLs, and J-V curves measured in the dark of champion for devices using e) PEDOT:PSS HTLs, f) PTAA HTLs.

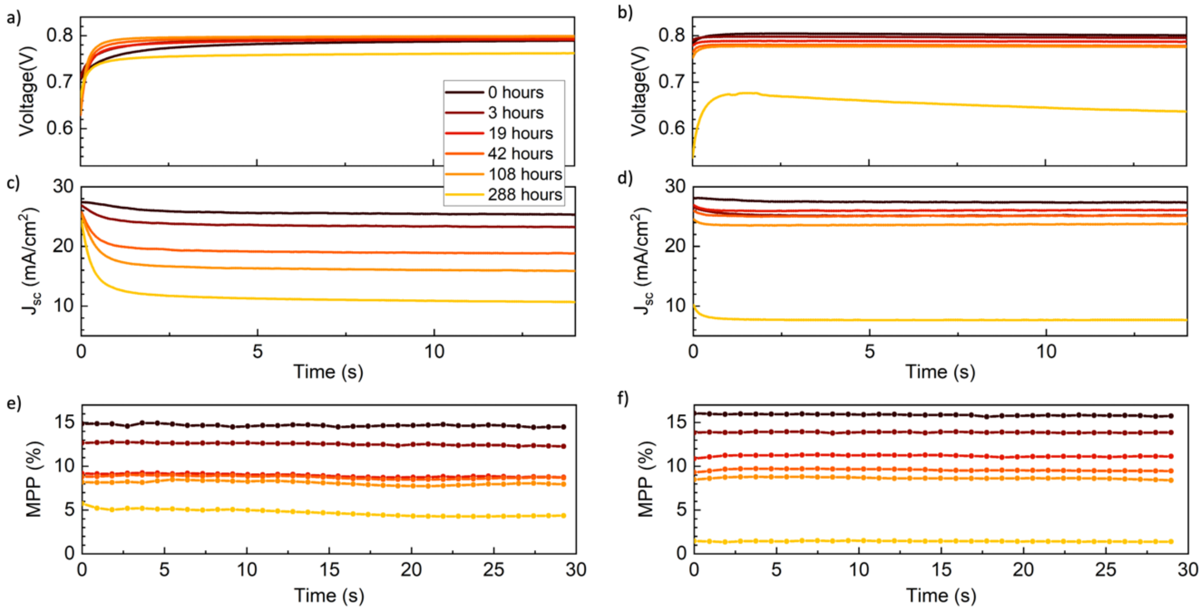

Figure S12. Voltage, current density and maximum power point (MPP) of champion ITO/HTL/ $FA_{0.83}Cs_{0.17}Pb_{0.5}Sn_{0.5}I_3$/PCBM/BCP/ Cr/Au solar cells over time after various periods of encapsulated aging under 65 °C and simulated full spectrum sun light (76 mW cm^{-2}) irradiance under open circuit conditions. Voltage at open circuit over time for devices using either a) PEDOT:PSS HTLs or b) PTAA HTLs, current density at short circuit over time for devices using either c) PEDOT:PSS HTLs or d) PTAA HTLs, and MPP over time for devices using either e) PEDOT:PSS HTLs or f) PTAA HTLs, each measured under AM1.5 100 mW cm^{-2} simulated sun light.

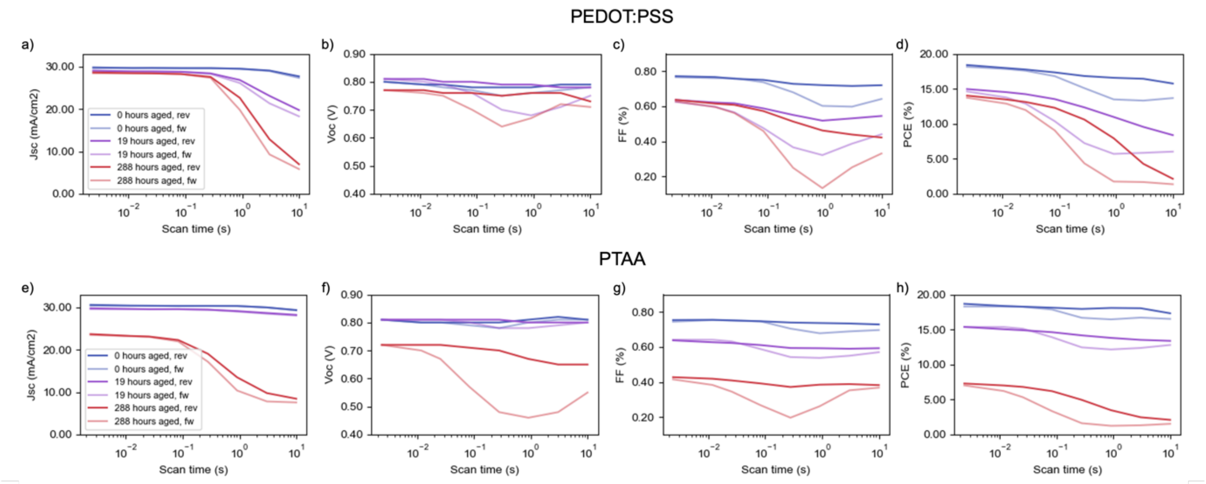

Figure S13. Parameters extracted from forwards and backwards J-V scans of lead-tin perovskite devices at varying rates, at multiple points during encapsulated aging under 65 °C and simulated full spectrum sun light (76 mW cm^{-2}) irradiance under open circuit conditions. a) Short-circuit current density, b) open-circuit voltage, c) fill factor, and d) power conversion efficiency for devices using a PEDOT:PSS HTL, and e) short-circuit current density, f) open-circuit voltage, g) fill factor, and h) power conversion efficiency for devices using a PTAA HTL.

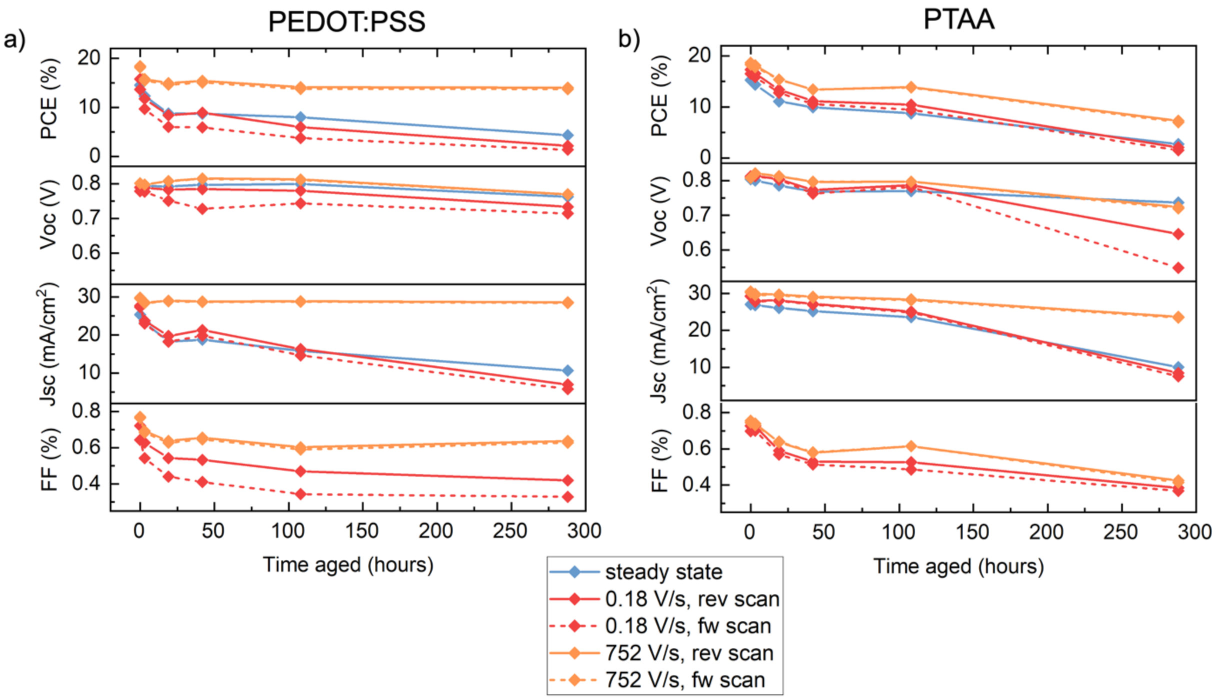

Figure S14. J-V parameters extracted from fast 752 V s^{-1} (orange) and slow 0.18 V s^{-1} (red) J-V scans of a champion ITO/HTL/$FA_{0.83}Cs_{0.17}Pb_{0.5}Sn_{0.5}I_3$/PCBM/BCP/Cr/Au device using a) PEDOT:PSS or b) PTAA HTLs. Solid lines represent parameters extracted from the initial scan decreasing in voltage, and dotted lines represent the subsequent scan increasing in voltage. Parameters from steady-state measurements of the same devices from figure 3a in the main text are overlaid in blue. Measurements were performed after various periods of encapsulated aging under 65 °C and simulated full spectrum sun light (76 mW cm^{-2}) irradiance under open circuit conditions.

Table S3. IonMonger J-V simulation parameters.

**Parameter**	**Symbol**	**Unit**	**Value**	**Comment**
**HTL (PEDOT:PSS)**				
Thickness	d	nm	30	From cross-sectional SEM
VB offset	Vb^{HLT}	eV	0.1	near match
Dielectric Permittivity	ϵ_r^{HTL}	-	2.46	[3]
VB density of states	g_{VB}^{HTL}	cm^{-3}	1×10^{20}	[4]
Hole mobility	μ_p^{HTL}	$cm^2\ V^{-1}\ s^{-1}$	1.5×10^{-2}	[5]
Doping density	p_0^{HTL}	cm^{-3}	5×10^{19}	Heavily p-doped[5]
Surface recombination velocity: electrons	S_n^{HTL}	$cm\ s^{-1}$	var	Informed from TRPL, tweaked to fit measured V_{oc}
**HTL (PTAA)**				
Thickness	d	nm	5	[6]
VB offset	χ^{HLT}	eV	0.1	[7]
Dielectric Permittivity	ϵ_r^{HTL}	-	3.5	[7]
VB density of states	g_{VB}^{HTL}	cm^{-3}	1×10^{20}	[7] Symmetric DOS
Hole mobility	μ_p^{HTL}	$cm^2\ V^{-1}\ s^{-1}$	4×10^{-5}	[7]
Shallow uniform acceptor density	p_0^{HTL}	cm^{-3}	5×10^{16}	[7] Slightly p-doped
Surface recombination velocity: Electrons	S_n^{HTL}	$cm\ s^{-1}$	var	Informed from TRPL, tweaked to fit measured V_{oc}
**Perovskite**				
Thickness	d	nm	660	From cross-sectional SEM
Bandgap	E_g^{AL}	eV	1.25	EQE data
Dielectric Permitivity	ϵ_r^{AL}	-	40	[5]
CB effective density of states	g_{CB}^{AL}	cm^{-3}	8.1×10^{18}	[5]
VB density of states	g_{VB}^{AL}	cm^{-3}	5.8×10^{18}	[5]
Electron mobility	μ_e^{AL}	$cm^2\ V^{-1}\ s^{-1}$	3.2	[5] raised slightly to fit data

Hole mobility	μ_p^{AL}	$cm^2\ V^{-1}\ s^{-1}$	3.2	[5] raised slightly to fit data
Lifetime	τ^{AL}	ns	var	Informed from TRPL
**ETL (PCBM)**				
Thickness	d	nm	20	From cross-sectional SEM
Offset	χ^{ELT}	eV	0.1	
Dielectric Permittivity	ϵ_r^{ETL}	-	5	[8]
CB effective density of states	g_{CB}^{ETL}	cm^{-3}	1×10^{20}	[8]
Electron mobility	μ_e^{ETL}	$cm^2\ V^{-1}\ s^{-1}$	2×10^{-3}	[8]
Shallow uniform donor density	n_0^{ETL}	cm^{-3}	5×10^{16}	[8]
Surface recombination velocity: holes	S_p^{ETL}	$cm\ s^{-1}$	var	Informed from TRPL, tweaked to fit measured V_{oc}

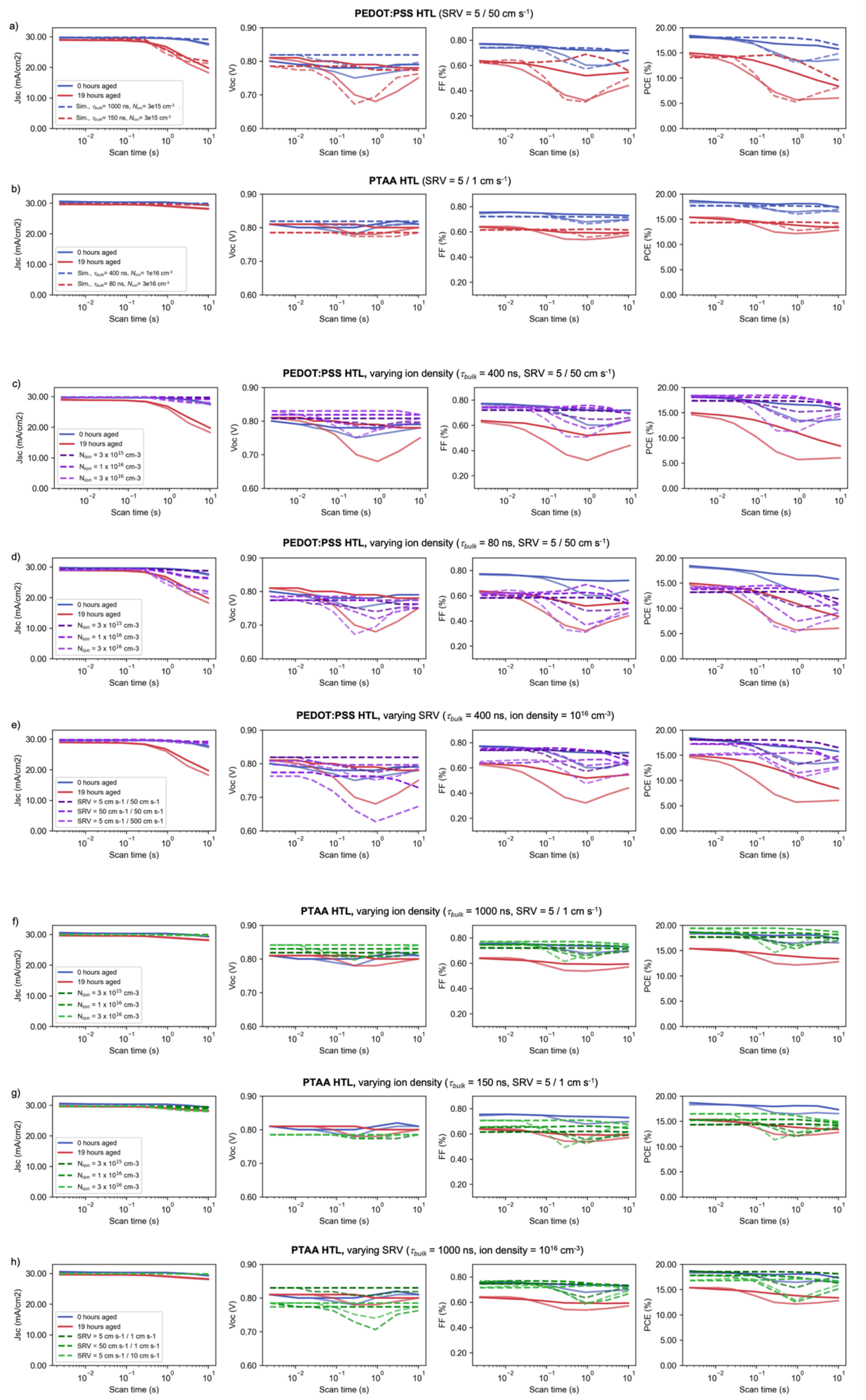

PEDOT:PSS HTL (SRV = 5 / 50 cm s-1)
a)
0 hours aged
19 hours aged
Jsc (mA/cm2)
Voc (V)
FF (%)
PCE (%)
Scan time (s)
PTAA HTL (SRV = 5 / 1 cm s-1)
b)
PEDOT:PSS HTL, varying ion density (τbulk = 400 ns, SRV = 5 / 50 cm s-1)
c)
PEDOT:PSS HTL, varying ion density (τbulk = 80 ns, SRV = 5 / 50 cm s-1)
d)
PEDOT:PSS HTL, varying SRV (τbulk = 400 ns, ion density = 1016 cm-3)
e)
SRV = 5 cm s-1 / 50 cm s-1
SRV = 50 cm s-1 / 50 cm s-1
SRV = 5 cm s-1 / 500 cm s-1
PTAA HTL, varying ion density (τbulk = 1000 ns, SRV = 5 / 1 cm s-1)
f)
PTAA HTL, varying ion density (τbulk = 150 ns, SRV = 5 / 1 cm s-1)
g)
PTAA HTL, varying SRV (τbulk = 1000 ns, ion density = 1016 cm-3)
h)
SRV = 5 cm s-1 / 1 cm s-1
SRV = 50 cm s-1 / 1 cm s-1
SRV = 5 cm s-1 / 10 cm s-1

Figure S15. Simulations of J-V parameters at varying scan rates using the IonMonger software,[5] overlaid on measured J-V parameters at varying scan rates after 0 and 19 hours of encapsulated aging under 65 °C and simulated full spectrum sun light (76 mW cm^{-2}) irradiance under open circuit conditions. Dark lines represent parameters extracted from the initial reverse scans (decreasing in voltage), and pale lines represent the subsequent forward scan (increasing in voltage). a) Simulated and measured J-V parameters at varying scan rates for devices using a PEDOT:PSS HTL, showing the simulations which yield the best fit to experimental data. b) Simulated and measured J-V parameters at varying scan rates for devices using a PTAA HTL, showing the simulations which yield the best fit to experimental data. c) Simulated and measured J-V parameters at varying scan rates for devices using a PEDOT:PSS HTL, showing simulations for a bulk lifetime of 400 ns and a varying ion density. d) Simulated and measured J-V parameters at varying scan rates for devices using a PEDOT:PSS HTL, showing simulations for a bulk lifetime of 80 ns and a varying ion density. e) Simulated and measured J-V parameters at varying scan rates for devices using a PEDOT:PSS HTL, showing simulations for a bulk lifetime of 400 ns and a varying perovskite/PEDOT:PSS surface recombination velocity. f) Simulated and measured J-V parameters at varying scan rates for devices using a PTAA HTL, showing simulations for a bulk lifetime of 1000 ns and a varying ion density. g) Simulated and measured J-V parameters at varying scan rates for devices using a PTAA HTL, showing simulations for a bulk lifetime of 150 ns and a varying ion density. h) Simulated and measured J-V parameters at varying scan rates for devices using a PTAA HTL, showing simulations for a bulk lifetime of 1000 ns and a varying perovskite/PTAA surface recombination velocity.

Table S4. SCAPS J-V simulation parameters.

Parameter	Symbol	Unit	Value	Comment
**HTL (PEDOT:PSS)**				
Thickness	d	nm	30	From cross-sectional SEM
Bandgap	E_g^{HTL}	eV	2.6	[2]
Electron Affinity	χ^{HLT}	eV	2.54	Near QFLS = V_{oc} match
Dielectric Permittivity	ϵ_r^{HTL}	-	2.46	[3]
CB effective density of states	g_{CB}^{HTL}	cm^{-3}	1×10^{20}	[4]
VB density of states	g_{VB}^{HTL}	cm^{-3}	1×10^{20}	Symmetric DOS
Electron mobility	μ_e^{HTL}	$cm^2 V^{-1} s^{-1}$	1.5×10^{-2}	[5]
Hole mobility	μ_p^{HTL}	$cm^2 V^{-1} s^{-1}$	1.5×10^{-2}	[5]
Shallow uniform donor density	n_0^{HTL}	cm^{-3}	1×10^5	Low value (p-doped)
Shallow uniform acceptor density	p_0^{HTL}	cm^{-3}	5×10^{19}	Heavily p-doped[5]
**HTL / Perovskite Interface (PEDOT:PSS)**				
Surface recombination velocity: electrons	S_n^{HTL}	$cm\ s^{-1}$	1000	Informed from TRPL, tweaked to fit measured V_{oc}
Surface recombination velocity: Holes	S_p^{HTL}	$cm\ s^{-1}$	1000	Informed from TRPL, tweaked to fit measured V_{oc}
**HTL (PTAA)**				
Thickness	d	nm	5	[6]
Bandgap	E_g^{HTL}	eV	3	[7]
Electron Affinity	χ^{HLT}	eV	2.14	[7]
Dielectric Permittivity	ϵ_r^{HTL}	-	3.5	[7]
CB effective density of states	g_{CB}^{HTL}	cm^{-3}	1×10^{20}	[7]
VB density of states	g_{VB}^{HTL}	cm^{-3}	1×10^{20}	[7] Symmetric DOS
Electron mobility	μ_e^{HTL}	$cm^2 V^{-1} s^{-1}$	1×10^{-5}	[7]

Hole mobility	μ_p^{HTL}	$cm^2\ V^{-1}\ s^{-1}$	1×10^{-5}	[7]
Shallow uniform donor density	n_0^{HTL}	cm^{-3}	1×10^{5}	Low value (p-doped)
Shallow uniform acceptor density	p_0^{HTL}	cm^{-3}	5×10^{16}	[7] Slightly p-doped
**HTL / Perovskite Interface (PTAA)**				
Surface recombination velocity: electrons	S_n^{HTL}	$cm\ s^{-1}$	1000	Informed from TRPL, tweaked to fit measured V_{oc}
Surface recombination velocity: Holes	S_p^{HTL}	$cm\ s^{-1}$	1000	Informed from TRPL, tweaked to fit measured V_{oc}
**Perovskite**				
Thickness	d	nm	660	From cross-sectional SEM
Bandgap	E_g^{AL}	eV	1.25	EQE data
Electron Affinity	χ^{AL}	eV	3.90	[5]
Dielectric Permitivity	ϵ_r^{AL}	-	40	[5]
CB effective density of states	g_{CB}^{AL}	cm^{-3}	2.2×10^{18}	[5]
VB density of states	g_{VB}^{AL}	cm^{-3}	2.2×10^{18}	[5]
Electron mobility	μ_e^{AL}	$cm^2\ V^{-1}\ s^{-1}$	5	[5] raised slightly to fit data
Hole mobility	μ_p^{AL}	$cm^2\ V^{-1}\ s^{-1}$	5	[5] raised slightly to fit data
Shallow uniform donor density	n_0^{AL}	cm^{-3}	1×10^{10}	[5]
Shallow uniform acceptor density	p_0^{AL}	cm^{-3}	1×10^{14}	Measured background carrier density
Neutral defect density lifetime	τ^{AL}	ns	2.5×10^{3}	Informed from TRPL
Single acceptor defect density	T_d^-	cm^{-3}	1×10^{14}	Same density as acceptor density
Electron/hole capture cross section for single acceptor defect	σ_n	cm^{-2}	1×10^{-17}	Tweaked for longer lifetime than neutral defects
**Perovskite / ETL Interface**				

Surface recombination velocity: electrons	S_n^{ETL}	cm s^{-1}	1000	Informed from TRPL, tweaked to fit measured V_{oc}
Surface recombination velocity: holes	S_p^{ETL}	cm s^{-1}	1000	Informed from TRPL, tweaked to fit measured V_{oc}
**ETL (PCBM)**				
Thickness	d	nm	20	From cross-sectional SEM
Bandgap	E_g^{ETL}	eV	2	[8]
Electron Affinity	χ^{ELT}	eV	3.9	[8]
Dielectric Permittivity	ϵ_r^{ETL}	-	5	[8]
CB effective density of states	g_{CB}^{ETL}	cm^{-3}	1 x 10^{20}	[8]
VB density of states	g_{VB}^{ETL}	cm^{-3}	1 x 10^{20}	[8]
Electron mobility	μ_e^{ETL}	cm^2 V^{-1} s^{-1}	2 x 10^{-3}	[8]
Hole mobility	μ_p^{ETL}	cm^2 V^{-1} s^{-1}	2 x 10^{-3}	[8]
Shallow uniform donor density	n_0^{ETL}	cm^{-3}	1 x 10^5	[8]
Shallow uniform acceptor density	p_0^{ETL}	cm^{-3}	1 x 10^5	[8]

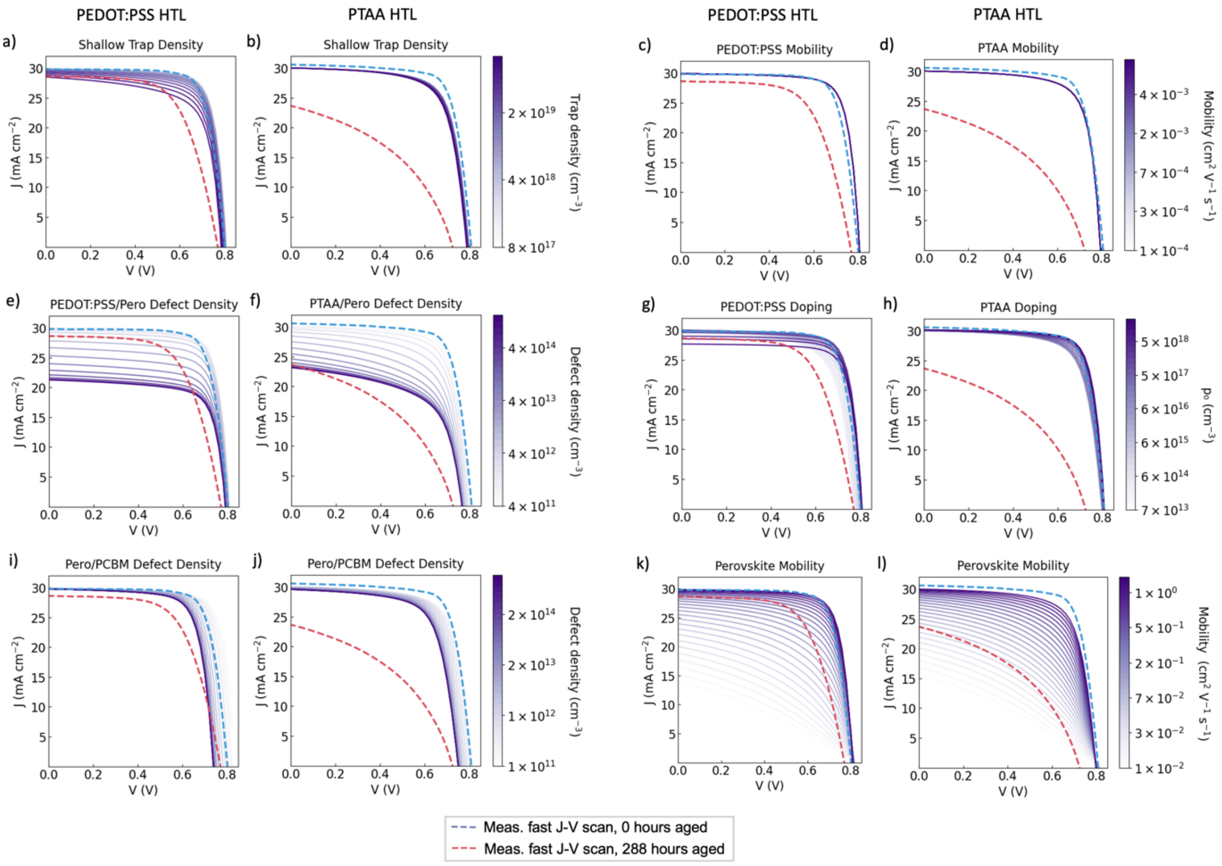

Figure S16. Simulated J-V curves of ITO/PEDOT:PSS or PTAA/ $FA_{0.83}Cs_{0.17}Pb_{0.5}Sn_{0.5}I_3$/PCBM/BCP/Cr/Au devices produced with SCAPS-1D[6] (full list of parameters in Table S4). J-V curves presented are simulated by varying shallow (0.03 eV from conduction band) electron trap density in the perovskite bulk for devices with a) a PEDOT:PSS or b) a PTAA HTL, mobility in the c) PEDOT:PSS or d) PTAA HTL, defect density at e) the perovskite/PEDOT:PSS or f) PTAA interface, acceptor density in g) the PEDOT:PSS or h) PTAA HTL, defect density at the perovskite/PCBM interface for devices with i) a PEDOT:PSS or j) a PTAA HTL, and electron and hole mobility in the perovskite absorber material for devices with k) a PEDOT:PSS or l) a PTAA HTL. Real device data is overlaid as dotted lines, showing fast (752 V s^{-1}) J-V scans of the champion devices when fresh and after 288 hours of aging.

Supplementary References

1. Carron, R. *et al.* Bandgap of thin film solar cell absorbers: A comparison of various determination methods. *Thin Solid Films* **669**, 482–486 (2019).
2. Lim, J. *et al.* Long-range charge carrier mobility in metal halide perovskite thin-films and single crystals via transient photo-conductivity. *Nat Commun* **13**, 4201 (2022).
3. Lim, V. J. -Y. *et al.* Air-Degradation Mechanisms in Mixed Lead-Tin Halide Perovskites for Solar Cells. *Advanced Energy Materials* 2200847 (2022) doi:10.1002/aenm.202200847.
4. Krückemeier, L., Liu, Z., Kirchartz, T. & Rau, U. Quantifying Charge Extraction and Recombination Using the Rise and Decay of the Transient Photovoltage of Perovskite Solar Cells. *Advanced Materials* **35**, 2300872 (2023).
5. Courtier, N. E., Cave, J. M., Walker, A. B., Richardson, G. & Foster, J. M. IonMonger: a free and fast planar perovskite solar cell simulator with coupled ion vacancy and charge carrier dynamics. *J Comput Electron* **18**, 1435–1449 (2019).
6. Burgelman, M., Nollet, P. & Degrave, S. Modelling polycrystalline semiconductor solar cells. *Thin Solid Films* **361–362**, 527–532 (2000).
