# Supplementary material for: Disentangling degradation pathways of narrow bandgap lead-tin perovskite material and photovoltaic devices
Source: Nat Commun. 2025 Jul 1;16:5450. doi: 10.1038/s41467-025-58489-9 (PMC12219749; doi:10.1038/s41467-025-58489-9)
Supplement: Supplementary file 2 — Reporting Summary [file 41467_2025_58489_MOESM2_ESM.pdf]

nature portfolio

Corresponding author(s): Henry J. Snaith

Solar Cells Reporting Summary

Nature Portfolio wishes to improve the reproducibility of the work that we publish. This form is intended for publication with all accepted papers reporting the characterization of photovoltaic devices and provides structure for consistency and transparency in reporting. Some list items might not apply to an individual manuscript, but all fields must be completed for clarity.

For further information on Nature Research policies, including our data availability policy, see Authors & Referees.

Experimental design

Please check the following details are reported in the manuscript, and provide a brief description or explanation where applicable.

1. Dimensions

Area of the tested solar cells

☒ Yes ☐ No

The aperture areas tested on each substrate were 0.25 cm3 (3 devices per substrate) and 1.00 cm3 (1 device per substrate). Actual device areas were 0.31 cm3 and 1.14 cm3, respectively.

Explain why this information is not reported/not relevant.

Method used to determine the device area

☐ Yes ☒ No

Provide a description of the method and state where this information can be found in the text.

Aperture manufacturer specifies minimum cut accuracy of 0.05 mm.

2. Current-voltage characterization

Current density-voltage (J-V) plots in both forward and backward direction

☒ Yes ☐ No

Supplementary information, figure S11 (for 2 champion devices).

Voltage scan conditions

☒ Yes ☐ No

Voltage was swept from 0.9 V to - 0.2 V and back at a rate of 0.61 V s-1.

Explain why this information is not reported/not relevant.

Test environment

☒ Yes ☐ No

Measurements were carried out on encapsulated devices in ambient conditions.

Explain why this information is not reported/not relevant.

Protocol for preconditioning of the device before its characterization

☐ Yes ☒ No

Provide a description of the protocol.

No explicit preconditioning was performed. Measurements were carried out in the following order: steady-state Voc, J-V scans, MPPT, steady-state Jsc.

Stability of the J-V characteristic

☒ Yes ☐ No

Steady-state Voc and Jsc measurements were carried out by holding devices at 0V or Voc for 30 seconds. Steady-state PCE measurements were carried out using an MPP tracker based on a gradient descent algorithm to measure to maximum power for 60 seconds. Traces of two champion devices provided in figure 12 of supplementary information.

Explain why this information is not reported/not relevant.

3. Hysteresis or any other unusual behaviour

Description of the unusual behaviour observed during the characterization

☒ Yes ☐ No

Hysteresis was extensively investigated by carrying out variable rate J-V scans (details in manuscript, section 2.4).

Explain why this information is not reported/not relevant.

Related experimental data

☒ Yes ☐ No

Section 2.4 and 2.5 of main text

Explain why this information is not reported/not relevant.

4. Efficiency

External quantum efficiency (EQE) or incident photons to current efficiency (IPCE)

☒ Yes ☐ No

EQE measurements of two champion devices are provided in figure S11 of supplementary information.

Explain why this information is not reported/not relevant.

A comparison between the integrated response under the standard reference spectrum and the response measure under the simulator

☒ Yes ☐ No

Integrated Jsc from EQE for two champion devices is also shown in figure S11 of supplementary information.

Explain why this information is not reported/not relevant.

For tandem solar cells, the bias illumination and bias voltage used for each subcell

☐ Yes ☒ No

Provide a description of the measurement conditions.

Devices tested were all single junctions.

5. Calibration

Light source and reference cell or sensor used for the characterization

☒ Yes ☐ No

Devices were measured under AM1.5G illumination (WaveLabs Sinus-220 solar simulator) with 100 mW cm-2 equivalent irradiance, certified by KG3-filtered Si reference photodiode.

Explain why this information is not reported/not relevant.

Confirmation that the reference cell was calibrated and certified

☒ Yes ☐ No

Fraunhofer ISE

Explain why this information is not reported/not relevant.

Calculation of spectral mismatch between the reference cell and the devices under test

☐ Yes ☒ No

Provide a value of the spectral mismatch and/or a description of how it has been taken into account in the measurements.

No spectral mismatch assumed (mismatch factor of 1) as Wavelabs spectrum very close to AM1.5.

6. Mask/aperture

Size of the mask/aperture used during testing

☒ Yes ☐ No

0.25 cm3 and 1.00 cm3

Explain why this information is not reported/not relevant.

Variation of the measured short-circuit current density with the mask/aperture area

☐ Yes ☒ No

Report the difference in the short-circuit current density values measured with the mask and aperture area.

This was not investigated.

7. Performance certification

Identity of the independent certification laboratory that confirmed the photovoltaic performance

☐ Yes ☒ No

Identify the independent certification laboratory.

Photovoltaic performance was not externally certified.

A copy of any certificate(s)

☐ Yes ☒ No

Certificate copies should be provided in the Supplementary information. Please state the supplementary item number.

Photovoltaic performance was not externally certified.

8. Statistics

Number of solar cells tested

☒ Yes ☐ No

4 substrates containing 4 devices each were tested (16 total).

Explain why this information is not reported/not relevant.

Statistical analysis of the device performance

☒ Yes ☐ No

Median, Q1 and Q3 of device performance is shown in figure 3 in the manuscript.

Explain why this information is not reported/not relevant.

9. Long-term stability analysis

Type of analysis, bias conditions and environmental conditions

☒ Yes ☐ No

Samples were encapsulated with a glass slide attached to the substrate with UV-activated epoxy which was cured for 3 minutes (Everlight Eversolar AB-341). For films, a recessed cavity glass with epoxy only deposited at the encapsulation edge was used to allow for optical measurements, and for devices the active area was fully covered by the epoxy. Before encapsulation, perovskite material was removed at the epoxy edge for optimal adhesion. Samples were aged in an ambient, illuminated aging chamber (Atlas Suntest XLS+) at 65 °C and simulated full spectrum sun light (76 mW cm-2) irradiance under open circuit conditions. Before any measurement was performed, samples were removed from the aging environment and allowed to come to room temperate without direct illumination for 20 minutes. Wide variety of analysis was carried out on aged samples (see manuscript).

Explain why this information is not reported/not relevant.
